# Perspectives of pain specialists, patients, and family members on long-term opioid use for chronic non-cancer pain: a qualitative study

**DOI:** 10.1186/s12871-021-01501-8

**Published:** 2021-11-09

**Authors:** Rattaphol Seangrung, Thongchai Tempeetikul, Supasit Pannarunothai, Supalak Sakdanuwatwong

**Affiliations:** 1 Department of Anesthesiology, Faculty of Medicine, Ramathibodi Hospital, Mahidol University, 270 Rama VI Road, Ratchathewi, Bangkok, 10400 Thailand; 2 Department of Anesthesiology, Maharat Nakhon Ratchasima Hospital, 49 Changpueak Road, Amphoe Mueang, Nakhon Ratchasima, 30000 Thailand; 3 Center for Health Equity Monitoring Foundation, 173/113, Moo 7, Phitsanulok-Nakhon Sawan Road, Thapho, Mueang District, Phitsanulok, 65000 Thailand

**Keywords:** Comment, Long term, Opioid use, Chronic non-cancer pain

## Abstract

**Background:**

Opioids are currently prescribed for chronic non-cancer pain (CNCP), and some patients use opioids continuously for long-term treatment. Stakeholders’ awareness about long-term opioid therapy is essential for improving the safety and effectiveness of pain treatment. The purpose of this study is to explore the perspectives of pain specialists, patients, and family caregivers about long-term opioid use in CNCP management.

**Methods:**

This study was a qualitative study and adhered to the COREQ guidelines. Pain specialists (*n* = 12), patients (*n* = 14), and family members (*n* = 9) were recruited to the study by purposive sampling at the Pain Clinic of Ramathibodi Hospital. Semi-structured interviews were recorded, verbatim transcribed, conceptually coded, and analyzed using Atlas.ti 8.0.

**Results:**

All groups of participants described opioids as non-first-line drugs for pain management. Opioids should be prescribed only for severe pain, when non-opioid pharmacotherapy and non-pharmacological therapies are not effective. Patients reported that the benefits of opioids were for pain relief, while physicians and most family members highlighted that opioid use should improve functional outcomes. Physicians and family members expressed concerns about opioid-related side effects, harm, and adverse events, while patients did not. Patients confirmed that they would continue using opioids for pain management under supervision. However, physicians stated that they would taper off or discontinue opioid therapy if patients’ pain relief or functional improvement was not achieved. Both patients and family members were willing to consider non-pharmacological therapies if potential benefits existed. Patient education, doctor–patient/family relationships, and opioid prescription policies were proposed to enhance CNCP management.

**Conclusion:**

Long-term opioid therapy for CNCP may be beneficial in patients who have established realistic treatment goals (for both pain relief and functional improvement) with their physicians. Regular monitoring and evaluation of the risks and benefits, adverse events, and drug-related aberrant behaviors are necessary. Integrated multimodal multidisciplinary therapies and family member collaborations are also important for improving CNCP management.

**Supplementary Information:**

The online version contains supplementary material available at 10.1186/s12871-021-01501-8.

## Background

Chronic non-cancer pain (CNCP) is defined as pain that persists for at least 3 months beyond tissue healing and is not related to malignant disease. CNCP interferes with patients’ daily activities and physical, emotional, and social functions, and has a severe negative impact on quality of life [1]. The prevalence rate of chronic pain is high in older people, at approximately 40 to 90% [2]. Thailand is currently facing an increase in the aging population; the third highest in Asia [3]. A 2013 study reported that Thailand had a high prevalence of CNCP: almost 20% of the working population [4]. The aims of treatment in these patients focus on reducing pain scores and improving function and quality of life. However, many patients continue to suffer from pain after receiving long-term non-opioid and non-pharmacological therapy [5]. Opioids are potent pain medications for moderate to severe pain management. The Centers for Disease Control and Prevention (CDC) recommends prescribing opioids for CNCP if the benefits of pain relief and functional improvement outweigh the harm [6]. Previous studies have reported that short-term opioid therapy is moderately effective for pain relief and small improvements in functional outcomes [7–12]. However, the prolonged use of opioids is associated with an increased risk of addiction, abuse, or dependence [9]. Recent evidence has shown an increase in the incidence and prevalence of long-term opioid therapy for CNCP, but studies of its efficacy and outcomes remain inconclusive [13, 14].

Many studies have reported that general physicians perceive multiple barriers to prescribing opioids in the long term [15]. Lack of knowledge and clinical skills regarding alternative treatments, as well as the fear of opioid-induced side effects, tolerance, and addiction, are the main problems for continuing therapy [15–19]. Nevertheless, many CNCP patients are adamant that opioids can control their pain, with little concern about adverse effects or abuse [20, 21]; these patients will need to continue taking opioids in the future [21]. However, the beliefs of physicians and CNCP patients may not be consistent. CNCP patients must collaborate with healthcare providers, as well as with family members, for their treatment. A pain specialist is a healthcare professional who serves as a consultant to other physicians and is the principal treating physician. In general, CNCP patients who are long-term users of opioid therapy are under the care of pain specialists. The present study is the first qualitative study to explore stakeholders’ perspectives about long-term opioid therapy, covering pain specialists, CNCP patients, and their family members.

## Methods

### Study design and setting

A qualitative, cross-sectional study was conducted in the form of semi-structured interviews with pain specialists, CNCP patients, and their family members at the Pain Clinic of Ramathibodi Hospital in Bangkok, Thailand and adhered to the COREQ guidelines.

### Ethical considerations

The protocol was approved (MURA2019/1198) on 27 November 2019 by the Ethics Committee (Chairman: Assistant Professor Dr. Chusak Okascharoen) of the Faculty of Medicine of Ramathibodi Hospital (Mahidol University, Bangkok, Thailand). Written informed consent was obtained from all participants according to the local regulations and to the principles of Helsinki Declaration. Prior to the interviews being conducted, participants were informed about the aim of the study and provided written informed consent. However, participants were informed that they could withdraw at any time from the study without any negative consequences. All data were confidential. Participants’ data were accessible only to the researchers who involved in the study and all document files were eradicated immediately following data analysis.

### Participants

The study was conducted from November 2019 until April 2020. We used purposive sampling to maximize the likelihood of recruiting participants with sufficient experience associated with long-term opioid therapy. Participants aged 18 and over who were fluent in the Thai language were informed about the study and provided their consent before participation. We recruited patients who 1) had CNCP for at least 3 months before attending the Pain Clinic; 2) had been prescribed with strong opioids (morphine, fentanyl, or methadone) for pain relief for more than 1 year in any dosage form, strength, or method of administration; and 3) came to follow-up. Family members who were caring and living with these CNCP patients for longer than 1 year were also included. In addition, pain specialists who had ever prescribed strong opioids to treat CNCP patients for more than 1 year were eligible to participate in our study.

### Data collection procedures

We created open-ended questions based on literature reviews, and a pilot tested them before use in this study. The questions explored the participants’ perspectives about long-term opioid use for the treatment of CNCP in relation to their personal context (Additional file 1). They covered the demographic data of participants, initiation of therapy with strong opioids, benefits of using opioids for long-term treatment, concerns about harm and adverse events, and plans for long-term treatment goals. Medical records were extracted for information about the cause of CNCP, opioids, and other pain medications prescribed. At the beginning of each interview, the interviewer introduced the objective of the study and explained the interviewing process. Both authors (TT and SS) conducted semi-structured interviews with the pain specialists, CNCP patients, and family members individually lasted 45 min per each session. All interviews were digitally audio-recorded and were then transcribed verbatim with participants’ permission. The interviews were conducted until thematic saturation was reached (no new themes and patterns emerged), with confirmation by the principal investigator (RS).

### Statistical analysis

The modified grounded theory was used to guide the coding and analyzing of transcription [22, 23]. All authors reviewed and carefully read the transcripts and coded individuals. After successive iterations of coding, the authors deliberated until they reached a complete consensus on the themes, and they then formatted distinct codebooks from the interviews of pain specialists, CNCP patients, and family members. The second author (TT) entered the coded interviews into Atlas.ti 8.0 [24].

## Results

An individual in-depth interview was conducted for approximately 30 to 45 min with each pain specialist, CNCP patient, and family member. Table 1 demonstrates the demographic characteristics of all participants. The analytical results of the contextual perspectives of long-term opioid use for CNCP are presented in Table 2.

**Table 1:** Demographic characteristics of all participants

Characteristic	Number (***n***)
**Pain specialists**	12
Age (years)	
< 40	6
> 40	6
Gender	
male	3
female	9
Working experience of pain (years)	
1–5	5
6–10	5
> 10	2
**Patients**	14
Age (years)	
18–40	5
41–60	8
> 60	1
Gender	
male	9
female	5
Duration of chronic pain (years)	
1–5	4
6–10	7
> 10	3
Duration of opioids therapy (years)	
1–5	11
6–10	1
> 10	2
**Family members**	9
Age (years)	
18–40	2
41–60	6
> 60	1
Gender	
male	2
female	7
Relationship to the patient	
parents/child	3
spouse	4
other	2

**Table 2:** Contextual perspectives of long-term opioid use for chronic non-cancer pain of participants

Contextual perspective	Participants mentioned: ***n*** (%)
**Pain specialists:** ***n*** **= 12**	
Specialist does not consider opioids to be first-line treatment	11 (92%)
Specialist considers alternative treatment before starting	6 (50%)
Specialist has concerns about multidisciplinary treatment	9 (75%)
Specialist has concerns about drug misuse	7 (58%)
Specialist reviews patient’s history and drug monitoring program	8 (66%)
Specialist evaluates benefits and harms of continued opioid therapy	5 (42%)
**Patients:** ***n*** **= 14**	
Patient agrees with opioid therapy	14 (100%)
Patient has concerns about potential consequences	4 (29%)
Patient has concerns about opioid side effects	2 (14%)
Patient experiences stigma	1 (7%)
Patient has no worries about opioid addiction	11 (78%)
Patient accepts opioids as treatment for long-term pain	10 (71%)
**Family members:** ***n*** **= 9**	
Family supports continued opioid use	4 (44%)
Family worries about opioid-related side effects	6 (66%)
Family has concerns about addiction	4 (44%)
Family detects opioid-related adverse events	3 (33%)
Family agrees with specialist’s decision	8 (89%)

### Pain specialists’ perspectives

Pain specialists discussed their experiences of prescribing strong opioids for CNCP patients within the domains of attitudes about prescribing opioids, benefits and effectiveness, concerns, and opioid prescribing policies.

#### Attitudes about prescribing opioids

Pain specialists reported that some patients have complex and severe chronic pain that is not responsive to non-opioids, pain intervention, physical therapy, or other alternative treatments. It is crucial to consider initiating and titrating opioids for pain control in these patients. However, pain specialists stated that they restricted opioid prescriptions as necessary, because opioids are not a first-line treatment for CNCP and have severe adverse effects. If the combination of appropriate opioid therapy with non-opioid and non-pharmacological treatments was able to improve both pain and function, then the specialists would initiate opioid therapy with close monitoring. As one specialist reported:

*“I prescribe opioids when patients are in severe pain and the primary treatment of choice—such as the maximum dose of non-opioid drugs, non-pharmacological treatment, or intervention treatment—is not effective for providing relief, and pain still interferes with their quality of life.”*

Some specialists expressed frustration and avoided opioid use. They mentioned that the mental and psychological health of CNCP patients should be discussed and evaluated before initiating opioid therapy. As one specialist said:

*“I avoid prescribing strong opioids for the treatment of these patients. I manage such pain using adjuvant drugs and interventions. Especially in the case of patients with an unidentified cause of pain, I always talk to the patients and organize interdisciplinary treatment with other specialists and psychiatrists.”*

Most specialists mentioned that they were comfortable prescribing opioids as a long-term treatment (longer than 1 year). However, they stated that drug monitoring and risk evaluation should be carefully assessed. They also mentioned that opioids should be provided for long-term pain control so that patients can perform their usual daily activities; this is considered long-term dosage management. As one specialist described:

*“There are not many patients who require opioids as a long-term treatment. Most of the patients are well-known cases. Most of them do not worry about reducing opioid dosage and are not willing to stop opioids. I think that the understanding of their pain and how to manage it is necessary. My treatment goals are achieving realistic pain relief and [ensuring patients are] able to participate in work and social activities.”*

#### Benefits and effectiveness

The benefits and effectiveness of long-term opioid therapy for CNCP remain unclear to pain specialists. Some patients required the continued use of the lowest effective dose of opioids to improve functional outcomes and daily activities, combined with non-opioid and non-pharmacological treatments. As one specialist said:

*“Well, you know, I have some patients who have received morphine for a long time, and they are able to do some more social activities, such as going to the mall or doing a few chores. However, medications may be a supplement to the treatment of pain, more or less. But I also have not given up on other treatments, such as psychotherapy. But not everyone is getting better.”*

Some pain specialists remarked that the cause of pain was a factor when deciding to initiate and continue prescribing opioids. If opioids were able to reduce pain, the specialists would calculate the total morphine milligram equivalents (MME) per day and convert to methadone if it was not contraindicated. As one specialist stated:

*“I have a patient who continues to have pain from a spinal cord injury [even] after titrating the maximal dose of anticonvulsants. So I tried to use morphine tablets to control his pain and calculated the total dosage converted to methadone. After he took methadone, he said the pain decreased and he slept better.”*

All specialists emphasized that, if the benefits did not outweigh the risks, they would talk with the patients to taper opioids and discontinue their use. They recommended additional non-pharmacological and non-opioid treatment options, or considered consulting a multidisciplinary working group if necessary. As three specialists remarked:

*“If I assessed that morphine did not help, I would advise patients to reduce or stop medication because drugs can't help them to improve. Continuing the medication will have more disadvantages.”*

*“I would advise patients to understand that morphine is not useful for pain control, but that other methods of treatment may be a better solution.”*

*“If morphine is not an appropriate treatment, I would advise patients to participate in treatment with specialists in various fields that can help with other rehabilitation procedures, or psychotherapies such as CBT [cognitive behavioral therapy].”*

#### Concerns

Most of the specialists reported concerns about the adverse effects of opioids. Drug monitoring and continued follow-ups should be scheduled every 2–3 months. As one specialist stated:

*“I am concerned about the possibility of opioid tolerance and misuse. I carefully assess [the patient’s] current effective dose, and cut back on their dose if possible.”*

Some specialists mentioned opioid use disorder. If they suspected this condition, they would offer or convert to methadone if it was not contraindicated. As one specialist said:

*“If there is any chance of drug addiction during treatment, I will avoid prescribing opioids at 90 MME per day. A small amount of opioids will be prescribed, and opioid rotation to methadone will be considered.”*

#### Opioid prescribing policies

Specialists reported that hospital policies, regulations, or clinical practice guidelines are necessary to promote the implementation of appropriate prescribing of opioids in CNCP patients. Overall, they believed that this would reduce the occurrence of opioid-related harm and prevent iatrogenic addiction. As some specialists said:

*“My hospital has a policy about prescribing opioids that helps us to easily manage [opioid prescriptions]. For example, it explicitly restricts prescribing more than 2 months.”*

*“I think that if the hospital has regulations that determine which doctor can prescribe morphine to chronic pain patients, we would be able to control the medication properly, and addiction would decrease.”*

All specialists stated that the implementation of education to healthcare providers and patients about opioid prescribing could help to reduce opioid use disorder. Moreover, they suggested that appropriate knowledge may promote more judicious opioid prescribing and ensure safer prescribing when indicated. As one specialist said:

*“I think that providing education about the advantages and disadvantages of long-term morphine use to patients and treating physicians was necessary. It decreased adverse events and harm.”*

Some specialists mentioned that the online national electronic medical record, which alerts opioid prescribing, might reduce patients’ opioid shopping or drug-seeking behaviors. Moreover, the electronic medical record helps to alert physicians about redundant drug prescribing. As some specialists mentioned:

*“I did not know whether patients had received morphine from other hospitals. Sometimes I felt discomfort about prescribing morphine. If I had known about all of the medications that my patient takes, I might have felt more confident to manage [their medications].”*

*“If I have ordered redundant drugs, the computer software alerts my prescriptions. This made me more confident, and reduced the mistakes made by people.”*

### Patients’ perspectives

CNCP patients mentioned their use of strong opioids for pain management for more than 1 year. The boundaries from the interviews were attitudes about using opioids, benefits and effectiveness, and concerns.

#### Attitudes about using opioids

Most patients rated their pain as the worst and reported disability caused by illness. However, patients accepted opioid therapy and expressed that opioids were useful for pain relief, and were better than previously prescribed non-opioid medications. As one patient said:

*“I have very bad pain and that pain really affects my life. I cannot walk, drive, work, or do any activities. I have tried many drugs but none provided good pain control. Morphine helps me for so many hours, more than other drugs”*

Some patients who disclosed the use of opioids for pain relief considered opioids to be the most effective medications for moderate to severe pain. They stated that their use of opioids was not for pleasure purposes. As one patient said:

*“I know that morphine is a strong drug, and I don’t worry about continuing opioid therapy because it provides better and fast-acting pain relief. Sure, it can relieve severe pain that other medications cannot. As long as it can ease my pain, I will continue using it. Of course, I don't use it for any purpose other than pain relief.”*

Some patients discussed tapering and discontinuing opioids if they lacked efficacy or had adverse events. They were ready to follow their pain specialists’ recommendations. As one patient said:

*“Drugs are prescribed to me depending on my pain level and the doctor’s decision. As the doctor said, if I had no response to pain control or I were harmed [by it], he would discontinue morphine. Whenever the doctor tells me to stop, I will stop.”*

#### Benefits and effectiveness

Most of the patients were comfortable to continue opioid therapy because of its effectiveness in pain relief. They also said that opioids encouraged the performance of their usual daily activities. As one patient said:

*“Today, I feel better when using morphine for acute pain relief. It allows me to help myself sometimes. Some nights I get pain and cannot sleep, and I use morphine to control my pain. That helps me a lot.”*

More than half of the patients agreed that opioid management has been much improved over all of pain. Furthermore, opioids were carefully prescribed by specialists under the drug monitoring program. These reasons explained why patients were confident to continue taking opioids. As one patient described:

*“Although morphine is a narcotic drug, it is prescribed with caution and well-controlled. I think that it has more benefits than risks. I feel no worries using it under the supervision of specialists.”*

#### Concerns

Overall, patients had no concerns about opioid side effects and did not experience any adverse events, psychosocial problems, or family relationship problems. As one patient mentioned:

*“I have no adverse experiences with morphine. I have no problems with moodiness, no trouble concentrating, no problems getting along with people. My family also agrees with me to continue morphine. They know that uncontrolled pain makes me annoyed, unhappy, and moody.”*

Some patients were concerned that long-term opioid therapy might be harmful for their organ function when combined with other medications. In this sense, they preferred to continue using opioids for pain control rather than other pain medications. As one patient said:

*“I worry about the side effects of taking many combined drugs. Do they have any side effects to my liver or my kidney? However, I can take some risks using pain medicines because I am not able to live my life with this pain level.”*

Patients with a history of alcoholism, smoking, or substance abuse reported slightly higher comfort with regards to continuing opioids without fear of opioid addiction. One patient described that:

*“Many people warn me to be careful when using morphine because I have a history of meth [amphetamine] addiction. However, in my experience, morphine is very different. I do not feel annoyed, terrible, tired, or drug-seeking when I take it. My mood is better, stable. I do not think that I can be addicted to it.”*

Alternatively, another patient relayed that:

*“I think that smoking or alcohol addictions are easier than morphine addiction. If we can stop smoking, we can stop using morphine too.”*

### Family members’ perspectives

Family members described their experiences of long-term opioid use in CNCP patients with respect to family relationships, attitudes about using opioids, benefits and effectiveness, and concerns.

#### Family relationships and attitudes about prescribing opioids

Most of the patients’ family members accepted their continued treatment with opioids because they perceived the severe pain level of the patients. They stated that opioids were able to resolve even the worst pain. They also mentioned that opioids improved daily physical activities and sleep. As one member said:

*“I know, whenever he [the patient] suffers severe pain, I let him try using this painkiller. I want his pain to be minimized. I witnessed him after he took it; he felt much better, he could rest, sometimes, he could walk. I think morphine is the key drug that can help him.”*

All members agreed that long-term opioid prescriptions were dependent on specialists’ decisions. They believed that strict compliance with the doctors’ recommendations would increase the safety and efficacy of treatment. One family member relayed that:

*“I don’t want him [the patient] to stop morphine. But I am careful not let him take more, and too often. If I notice some adverse events, I will take him to see the doctor.”*

Alternatively, one member stated that:

*“I do not interfere with the doctor’s prescription. The decision about a morphine prescription is dependent on the doctor’s opinion. I do not worry about continuing or stopping.”*

#### Benefits and effectiveness

Most family members approved the use of long-term opioids because of their effectiveness for pain control. They were able to accept the low level of adverse events. However, they noted that adverse events should be continuously monitored. One member expressed that:

*“I used to worry at his [the patient’s] first time using morphine, but I saw that it provided control over his pain. I saw some drowsiness and bit of confusion after use, but that was not much different from normal. He slept better. There are not many side effects so I will keep observing.”*

Another member mentioned that:

*“Morphine could provide some relief but he [the patient] still had pain. However, adequate use makes it easier for him to do more activities and sleep. Sometimes, I think that I see some confusion and personality changes, but it is not a big problem. This problem can be reduced and controlled.”*

Some of the family members mentioned that sleep disruption is a particularly frequent issue for chronic pain patients. As one member described, opioids are a medication that can be used as a sleep aid to take away pain.

*“Well, you know, every night she [the patient] lies down with pain. She cannot sleep and is agitated. Then she takes a pill, she feels a little bit well and sleeps well. Okay! I just say it helps control her pain.”*

#### Concerns

Most of the family members were concerned about the adverse effects of opioids. The primary concerns were opioid addiction and having psychosocial problems. The secondary concern was physical problems related to the use of opioids. As one family member noted:

*“When [the patient] started using morphine, I was so worried about addiction, withdrawal symptoms, and also emotional problems, which led to quarrels in my family.”*

Another described:

*“I am worried that morphine may cause adverse effects to his [the patient’s] health. He takes a lot of medications to control his underlying disease and he also has a problem with his kidneys.”*

## Discussion

This study is the first qualitative study to explore the perspectives of pain specialists, patients, and family members about long-term opioid use (for more than 1 year) for CNCP in Thailand. Each group of participants expressed various attitudes toward starting and continuing long-term opioid therapy. The treatment goals of opioid use were different between the groups in our study. Pain specialists mostly prescribed opioids to improve functional outcomes rather than to reduce pain as patients needed, following the CDC guidelines for prescribing opioids for chronic pain [6]. Pain specialists especially emphasized that multimodal and multidisciplinary therapies were more effective for chronic pain treatments than single modalities, as previous studies have reported [25–27]. Non-opioid pharmacological options, including acetaminophen, non-steroidal anti-inflammatory drugs, and anticonvulsants and antidepressants with analgesic properties, may provide pain relief for many types of CNCP [28–32]. Additionally, cognitive behavioral therapy, exercise therapy, and pain intervention can promote the effectiveness of pharmacological therapies for increasing abilities, reducing pain, improving well-being, and decreasing catastrophic thinking [33–36].

CNCP patients usually experience irritability caused by pain, and opioids may allow them to relax. Family members regarded opioid use as beneficial to patients’ pain control and for improving family relationships. However, patients mainly asserted that opioids provided them with maximal pain relief and induced sleep. These results are consistent with previous research showing that CNCP patients need opioids for both pain relief and rest. Patients believe that opioids help them to control their pain, and that their pain will be severe if they do not have access to opioids [19, 21].

Another interesting finding was that our patients confirmed that they wished to continue long-term opioid therapy, although its long-term benefits and effectiveness were small. It is possible that chronic pain patients’ belief in opioids is valuable for a variety of pain control, despite their reported ongoing pain. In contrast, pain specialists stated that they would discontinue opioids if the benefits did not outweigh the risks, or would prescribe the lowest effective dosage to improve patients’ physical functions. Our study revealed that pain specialists preferred methadone over other opioids for long-term therapy, if its effectiveness was shown to be the same as that of previous studies [37–40]. Methadone possesses several useful properties; it has affinity to opioid receptors and is an antagonist of both N-methyl-D-aspartate receptors and serotonin–norepinephrine reuptake inhibitors, resulting in its use for treating back pain, neuropathic pain, chronic headache, and complex regional pain syndrome.

In our study, patients were more concerned with the potential achievement of maximal pain relief than with opioid-related harm. Similar to a previous survey of the attitudes and beliefs associated with long-term opioid use prescribed in CNCP, most patients were not very concerned about addiction or side effects [21]. CNCP affects physical functions and triggers emotional responses, including sleeplessness and social isolation. Therefore, the expectation of maximal pain relief is a primary target. Suboptimal physical activity and engagement, as well as a lack of meaning and pleasure in life, may also lead to increased levels of anxiety and depression, and an increased risk of opioid use disorder. However, the patients in our study talked about receiving appropriate close follow-up and clinical monitoring under the supervision of their physicians. Likewise, although pain specialists and family members had some concerns about drug misuse and opioid-related social problems, they also strongly agreed that appropriate follow-up and opioid dosage monitoring programs were necessary to control each patient’s current dosage, especially in cases of high risk of addiction.

In the present study, pain specialists proposed that the provision of adequate education to healthcare providers, patients, and other stakeholders would help to create a better understanding of opioid use in CNCP. Previous research has noted that good pain education is the key to improving pain management [15, 41]. Patients and their family members often have doubts about both pain and treatment plans. Thus, adequate education and information aimed at helping them to understand pain conditions is likely to reduce anxiety, decrease the misuse of pain medications, and increase patient satisfaction. In a recent report, deploying pain education strategies in conjunction with physical therapy led to a decrease in pain and disability [42]. Furthermore, training patients to use pain self-management strategies was able to significantly reduce long-term opioid use in patients with chronic pain [43]. Enhanced continuing pain education is therefore essential for health care professionals, to fill gaps in their knowledge and increase competencies related to pain management.

Finally, in the present study we also found that hospital regulations and policies can support the process of care. The pain specialists proposed the use of an online national electronic medical record that provides alerts for opioid prescribing, which they believe would control and prevent redundant opioid dispensing and reduce opioid shopping or drug-seeking behaviors. These results warrant consideration, because a previous report from Tasmania suggested that a real-time prescription monitoring program, DORA (Drugs and Poisons Information System Online Remote Access), is useful for clinical decision-making and reducing doctor shopping, and allows for better control of substances such as opioids [44, 45].

## Limitations

This study has some limitations. First, the study was only performed at a single institution; therefore, our results may not be generalizable to other situations. Second, our patients’ characteristics were not grouped by cause of pain, psychological disorder, pain interfering with daily activities and functions, occupational status, or type of opioid use. Finally, our study was conducted in Thailand and the results may not be generalizable to other races or nationalities because of the influence of sociocultural differences on beliefs and attitudes. Future research should assess opioid use disorder combined with interviewing for the evaluation of evidence.

## Conclusions

The benefits and effectiveness of long-term strong opioids prescribed for CNCP are limited. Opioids may be appropriate for only a small proportion of the causes of pain. Awareness about balancing the benefits against the possible side effects of long-term opioid use is recommended for its close monitoring in prolonged use. Diverse perspectives about long-term opioid use need to achieve consensus between professionals, patients, and family members, with appropriate education, sharing of information, well-planned and -organized treatment regimes, and acceptable healthcare environments. Concurrently, the treatment of CNCP warrants a multidisciplinary approach, including both pharmacological and non-pharmacological treatments.

## Supplementary Information

**Additional file 1.**

## Data Availability

The recordings and transcriptions of interviews are not openly available to conserve the confidential information of participants. All document files were eradicated immediately following data analysis. Themes and quotes from the data analysis are available in Thai. These data can be obtained by application to the Ethical Committee of the Faculty of Medicine, Ramathibodi Hospital. To apply, please contact the corresponding author at rattaphol_nu@hotmail.com, who will facilitate this process.

## Abbreviations

CNCP: Chronic non-cancer pain
CDC: Centers for Disease Control and Prevention
DORA: Drugs and Poisons Information System Online Remote Access
