# Supplementary material for: Perspectives of pain specialists, patients, and family members on long-term opioid use for chronic non-cancer pain: a qualitative study
Source: BMC Anesthesiol. 2021 Nov 9;21:275. doi: 10.1186/s12871-021-01501-8 (PMC8576950; doi:10.1186/s12871-021-01501-8)
Supplement: Supplementary file 1 — Additional file 1. [file 12871_2021_1501_MOESM1_ESM.docx]

**Additional file 1**

The open-ended questions used to ask about perspectives of long-term opioid use for chronic non-cancer pain

**Part I Questions for pain specialist**

1.1 How old are you?

1.2 How long have you practiced in a pain clinic?

1.3 Could you explain your considerations for prescribing opioids for chronic non-cancer pain patients?

1.4 Could you explain your opinions about long-term (> 1 year) opioid therapy in chronic non-cancer pain patients?

- 1. What are your treatment goals for the treatment of chronic non-cancer pain patients?

**Part II Questions for chronic non-cancer pain patients**

2.1 How old are you?

2.2 How long have you been in chronic pain? And what is the cause of your chronic pain?

2.3 How long have you been prescribed strong opioids for relieving chronic pain?

2.4 Could you explain your opinions when the doctor prescribed strong opioids as a treatment for your chronic pain?

2.5 Could you explain your opinions after receiving strong opioids for relieving your chronic pain for more than 1 year?

- 1. What are your goals for the treatment of chronic non-cancer pain?

**Part III Questions for family members**

3.1 How old are you?

3.2 What is your relationship with the patient?

3.3 How long have you taken care of the chronic non-cancer pain patient?

3.4 How long have you known that the patient has been prescribed strong opioids as a treatment for chronic pain?

3.5 Could you explain your opinions when the doctor prescribed strong opioids as a treatment for the patient’s pain?

3.6 Could you explain your opinions after the patient had received strong opioids for relieving chronic pain for more than 1 year?

3.7 What are your goals for the treatment of chronic non-cancer pain of the patient?
